# Collapsed Reticular Network and its Possible Mechanism during the Initiation and/or Progression of Hepatic Fibrosis

**DOI:** 10.1038/srep35426

**Published:** 2016-10-14

**Authors:** Shi-Lei Wen, Shi Feng, Shi-Hang Tang, Jin-Hang Gao, Lin-hao Zhang, Huan Tong, Zhao-Ping Yan, Ding Zhi Fang

**Affiliations:** 1 Department of Human Anatomy, West China School of Preclinical and Forensic Medicine, Sichuan University, Chengdu 610041, Sichuan, PR China; 2 Department of Gastroenterology, West China Hospital, Sichuan University, Chengdu 610041, Sichuan, PR China; 3 Department of Peptides Related to Human Diseases, State Key Laboratory of Biotherapy, Sichuan University, Chengdu 610041, Sichuan, PR China; 4 West China School of Medicine, Sichuan University, Chengdu 610041, Sichuan, PR China; 5 Department of Biochemistry and Molecular Biology, West China School of Preclinical and Forensic Medicine, Sichuan University, Chengdu 610041, Sichuan, PR China

## Abstract

Among the researches on hepatic fibrosis, great attention was paid to both hepatocytes and extracellular matrix (ECM). However, little focus was drawn on reticular fibrous network, which is important for demarcation and support of hepatocytes. The aim of this study was to investigate the change pattern of reticular fibers in hepatic fibrosis/cirrhosis and its underlying mechanism. In this study, thioacetamide (TAA) and bile duct ligation (BDL) were utilized to induce rat hepatic fibrosis respectively, and Human liver cirrhotic microassay was analyzed with IHC to confirm the results in animal experiment and to detect the metalloproteinases (MMPs) expressions. As a result, the reticular fibers decreased markedly after 1 week in TAA and 1 day in BDL treated rats. Multiple representative regulators of MMPs and MMPs increased significantly in their expressions and activities. Further more, in human liver cirrhotic microassay, MMPs expressions also showed similar patterns as that of animal experiment. In Conclusions: Degradation or collapse of reticular fibers in hepatic sinusoid can be considered as a pathological feature during the initiation and/or progression of hepatic fibrosis. Moreover, such degradation is associated with and probably caused by the over/dysregulated expression of MMPs.

The Extracellular Matrix (ECM) within the Disse space consists of a layer of basement membrane and fibrillar ECM molecules^1^. Reticular fibers, one of important component of ECM, branch and anastomose as a fibrous network delineating the space of Disse and forming a scaffold for the hepatocytes^2^. They work as a distinct structural unit for demarcation and support of the cellular components^3^,^4^. Numerous researches demonstrated that the basement membrane undergoes a destruction procedure during the progression of hepatic fibrosis^5^,^6^,^7^. However, very little attention was paid to the role of reticular fibers during that procedure, except for their greatly increased amount^8^,^9^. Very few reports on damaged reticular fibers in hepatic sinusoid were just pathological descriptions on liver cancer and fatty liver^10^,^11^,^12^, instead of on hepatic fibrosis. Thus, considering that the reticular fibers are supporting the hepatic lobule, exploring the role of reticular fibers, their change pattern during hepatic fibrosis might be an interesting phenomenon to facilitate the understanding of the pathological mechanism of hepatic fibrosis/cirrhosis.

Under normal status, ECM is in a certain dynamic balance of synthesis and breakdown, but such balance is upset during hepatic fibrosis when the liver suffers from chronic injury - both the character and amount of ECM could be changed^13^. It is well-known that matrix metalloproteinases (MMPs) and tissue inhibitors of metalloproteinases (TIMPs) are the main regulators of ECM during hepatic fibrosis^5^. In addition, the reticular fibers are mainly composed of collagen type III in association with other types of collagen (e.g., collagen type V), glycoproteins, and proteoglycans/glycosaminoglycans^3^,^4^. Among MMPs, the MMP-2 and MMP-9 can breakdown many components of ECM including collagen type III^14^,^15^, whereas TIMP-1 can reversibly bind to pro-MMP-9 and MMP-2 to deactivate them. Since that, in order to find out the mechanism underlying the change of reticular fibers, the activity and expression of MMP-2, MMP-9 and TIMP-1 were detected.

Meanwhile, the expressions and activities of MMPs are regulated by several different pathways and factors^16^,^17^, such as nitric oxide (NO), activator protein 1 (AP-1), nuclear factor kappa B (NF-κB), transforming growth factor-β1 (TGF-β1), and inflammatory cytokines. Therefore, some related representative factors were investigated in this research in order to explore the potential upstream regulators affecting MMPs.

## Results

### Hepatic fibrosis evaluation

**Figure 1:**
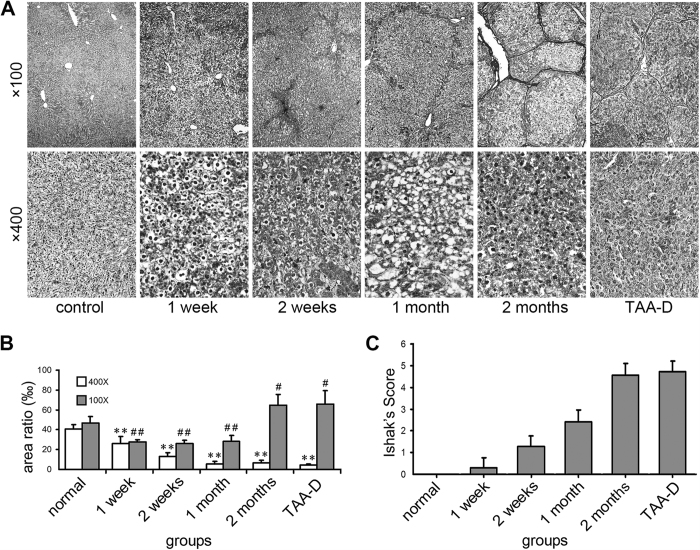
Change of reticular fibers and Ishak’s socre of TAA-treated rat liver tissues. (**A**) Reticular fiber stain (Gomori’s reticular stain, ×100 and ×400 magnifications, for inserts, the corresponding background was erased); (**B**) Areas of reticular fibers, n = 6/group; (**C**) Ishak’s socre. ×400: *p < 0.05, **p < 0.01 *vs.* control group; ×100: ^#^*p* < 0.05, ^##^*p* < 0.01 *vs.* control group.

**Figure 2:**
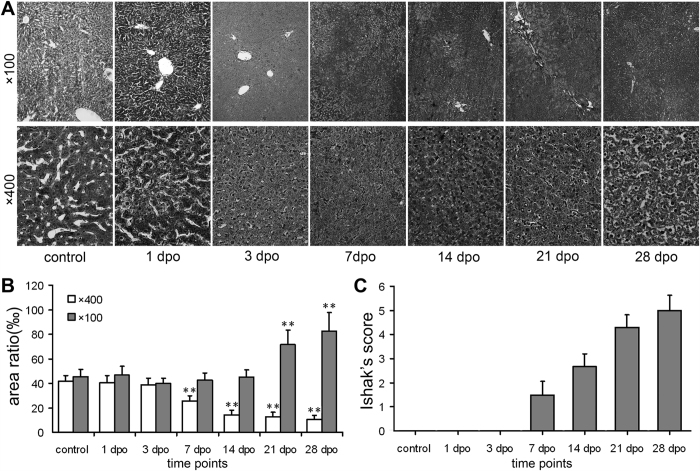
Change of reticular fibers and Ishak’s socre of BDL rat liver tissues. (**A**) Reticular fiber stain (Gomori’s reticular stain, ×100 and ×400 magnifications, for inserts, the corresponding background was erased); (**B**) Areas of reticular fibers; (**C**) Ishak’s socre. n = 6; ×400: *p < 0.05, **p < 0.01 *vs.* control group; ×100: ^#^*p* < 0.05, ^##^*p* < 0.01 *vs.* control group.

Liver sections were stained with haematoxylin and eosin (HE) and Sirius red to evaluate fibrotic level (Supplementary Figure S1). According to Ishak’s score system, thioacetamide (TAA) induced hepatic fibrosis developed gradually in association with drug administration time. The marked fibrosis appeared at 1 month after TAA administration and lasted to the last time point (Fig. 1C), while the typical fibrosis showed up at 21 dpo of the and bile duct ligation (BDL) group (Fig. 2C). Such pattern was similar to previous reports^18^,^19^.

### The change of reticular fibers in rat liver tissues

No significant difference was found among control rats time-matched for each TAA-treated rat (Supplementary Figure S2A,B). Reticular fiber stain for TAA treatment was shown in Fig 1A,B. ×100 and ×400 magnifications were captured (In Supplementary Figure S3, the corresponding background was erased, showing reticular fibers only, and therefore, the diversity of reticular fibers was more direct-viewing). The former was used to evaluate the global area of reticular fibers, and the latter was for assessment of the area of reticular fibers that located along the hepatic cords. The global area of reticular fibers of TAA group decreased markedly at the initial 3 time points, that are, 1 week, 2 weeks and 1 month (27.85 ± 1.85‰ *vs.* 44.55 ± 5.33‰, 26.30 ± 3.30‰ *vs.* 46.71 ± 6.03‰, 28.25 ± 6.16‰ *vs.* 46.97 ± 6.25‰, respectively; *p* < 0.05 or *p* < 0.01), while, inversely, such area enlarged significantly at the later 2 time points, known as 2 months and 3 months (TAA-D) (64.61 ± 10.93 *vs.* 44.54 ± 6.6‰ and 65.82 ± 13.54‰ *vs.* 45.54 ± 5.75‰, respectively; *p* < 0.05). On the other hand, the change pattern of reticular fiber area along the hepatic cords was another story: a consistent decrease of reticular fibers in TAA group was revealed throughout all the time points, which were 1 week, 2 weeks, 1 month and 2 months (26.09 ± 6.84‰ *vs.* 41.44 ± 5.23‰, 12.77 ± 4.06‰ *vs.* 43.49 ± 5.68‰, 5.6 ± 2.75‰ *vs.* 42.81 ± 6.5‰, 6.45 ± 2.53‰ *vs.* 43.05 ± 5.12‰, respectively; *p* < 0.01). Further more, the reticular fibers of TAA-D group remained at a low level even after TAA was withdrawn for a month, compared with control group (4.41 ± 1.07‰ *vs.* 40.49 ± 4.71‰; *p* < 0.01).

Reticular fiber stain for BDL group was presented in Fig. 2A,B. Global reticular fiber area had no difference from 1 dpo to 14 dpo (*p*>0.05), while significant increase of reticular fibers was noticed at 21 dpo and 28 dpo (71.65 ± 11.85‰ *vs.* 45.65 ± 6.64‰, 82.55 ± 15.32‰ *vs.* 43.07 ± 5.61‰, respectively; *p* < 0.05 or *p* < 0.01). Area of reticular fibers along hepatic cords showed continuous decrease from 7 dpo to 28 dpo (25.73 ± 4.2‰ *vs.* 41.89 ± 4.23‰, 14.4 ± 3.58‰ *vs.* 43.07 ± 5.61‰, 12.6 ± 4.01‰ *vs.* 42.28 ± 5.96‰, 10.72 ± 3.22‰ *vs.* 43.06 ± 4.8‰, respectively; *p* < 0.05 or *p* < 0.01).

### The change of collagen III in TAA-treated rat liver tissues

**Figure 3:**
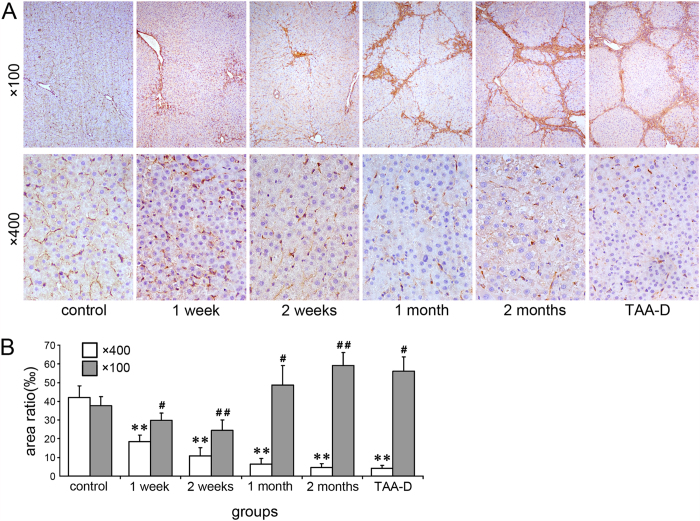
Change of collagen III in TAA-treated rat liver tissues. (**A**) Reticular fiber stain (Gomori’s reticular stain, ×100 and ×400 magnifications, for inserts, the corresponding background was erased); (**B**) Areas of collagen III positive staining, n = 5; ×400: *p < 0.05, **p < 0.01 *vs.* control group; ×100: ^#^*p* < 0.05, ^##^*p* < 0.01 *vs.* control group.

In order to confirm the results of the reticular fiber stain, immunohistochemistry (IHC) was performed to detect the expression of collagen III and pictures were captured in ×100 and ×400 magnifications (Fig. 3A). No significant difference was found among control rats time-matched for each TAA-treated rat (Supplementary Figure S2C,D). For the global area under magnification of ×100, compared with control, collagen III decreased at 1 week and 2 weeks but then increased at 1 month, 2 months and TAA-D, though significant difference was only observed in time points of 2 weeks, 2 months and TAA-D (Fig. 3B, *p* < 0.05 or *p* < 0.01). But under magnification of ×400, collagen III decreased significantly throughout the time points (Fig. 3B, *p* < 0.01).

### Expression of MMP-2, MMP-9 and TIMP-1 in rat and human livers

**Figure 4:**
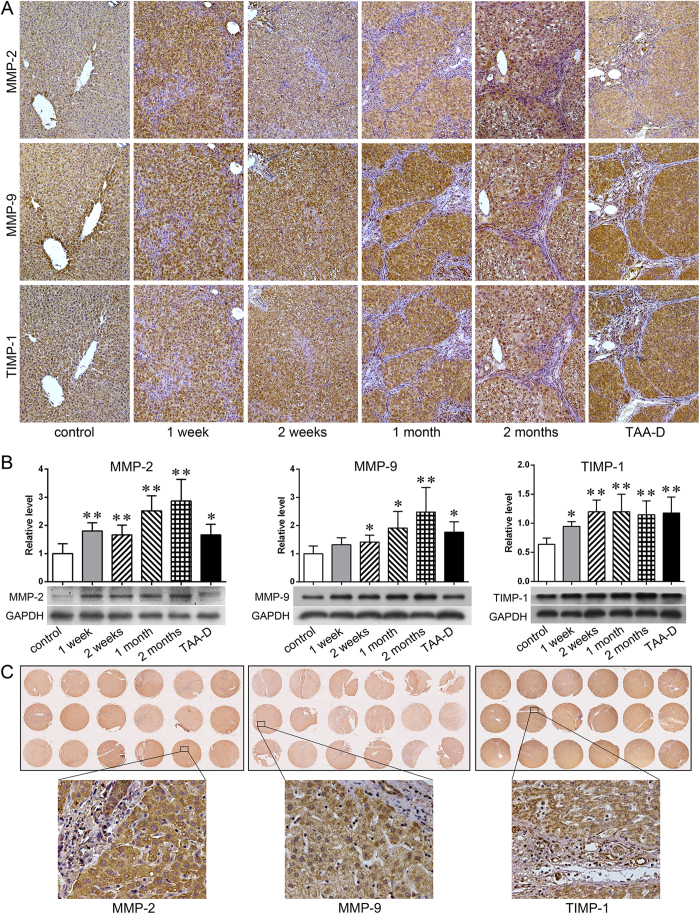
Expression of MMP-2, MMP-9 and TIMP-1 in rat and human livers. (**A**) IHC results of MMP-2, MMP-9 and TIMP-1 in rat liver tissues (×100 magnifications); (**B**) Western blot result of MMP-2, MMP-9 and TIMP-1 in rat liver tissues (n = 5, *p < 0.05, **p < 0.01 *vs.* control group); (**C**) IHC of MMP-2, MMP-9 and TIMP-1 in human liver tissue mircoarrays (×200 magnifications).

IHC and western blot were carried out to test TAA-treated rats (Fig. 4A). As IHC results showed, rare positive cells stained with MMP-2, MMP-9 and TIMP-1 were observed in the liver tissues of control rats time-matched for each TAA-treated rat (Supplementary Figure S4). However, their expressions increased greatly in TAA-treated rats, and the positive staining was mainly inside of the hepatocytes and cholangiocytes. Quantitatively, the protein levels of MMP-2, MMP-9 and TIMP-1 in TAA-treated rat liver tissues were significantly higher than these in the control rats, especially on the later time points (Fig. 4B. TAA group *vs.* control group, *p* < 0.05 or *p* < 0.01).

The expressions of MMP-2, MMP-9 and TIMP-1 in human cirrhotic liver tissue mircoarrays were also detected to check whether the expression patterns were similar to that of the animal experiment. The finding was that in human cirrhotic liver tissues, those three factors were all overexpressed as they were in TAA-treated rats (Fig. 4C).

### The activities of MMP-2 and MMP-9 in TAA-treated rat liver tissues

**Figure 5:**
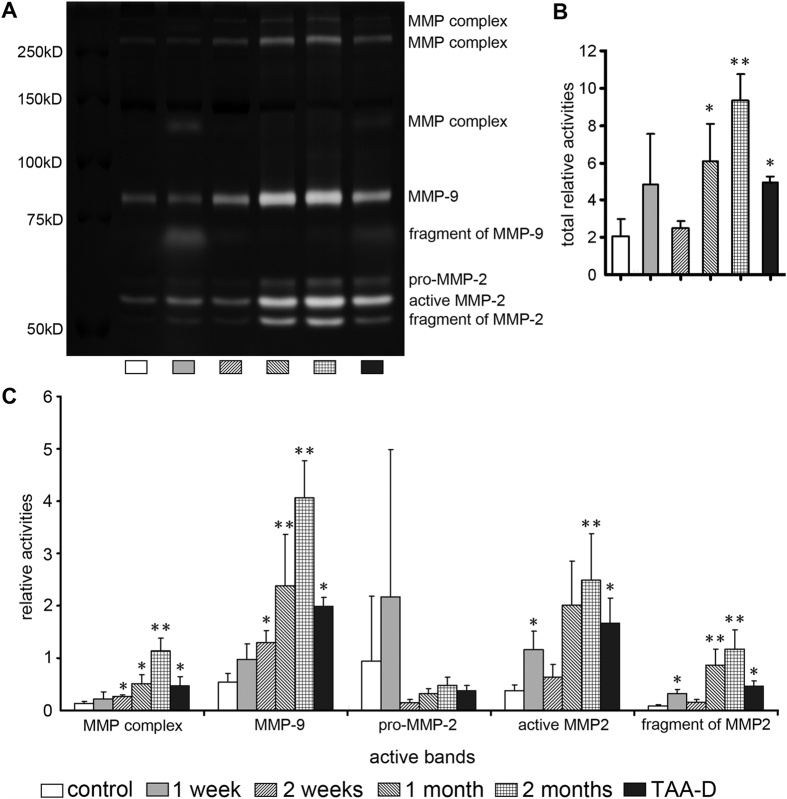
Activities of MMP2 and MMP-9 in TAA-treated rat liver tissues. (**A**) Activities of MMP-2 and MMP-9 by gelatin zymography; (**B**) Relative activities of total MMPs; (**C**) Relative activities of MMP complex, MMP-9, pro-MMP-2, active MMP-2 and fragment of MMP-2. n = 5; *p < 0.05, **p < 0.01 *vs.* control group.

In order to indirectly evaluate their degradation effects on reticular fibers, the activities of MMP-2 and MMP-9 in liver tissues were detected by performing gelatin zymography (Fig. 5A). Seven positive bands were obtained: MMP complex (three bands), MMP-9, pro-MMP-2, active MMP-2 and fragment of MMP-2. No significant difference was found among control rats time-matched for each TAA-treated rat (Supplementary Figure S4). The relative activities of MMP complex, MMP-9, int-MMP-2 and active MMP-2 increased obviously at each time point, in particular at 1 month and 2 months (Fig. 5C. TAA group *vs.* control group, *p* < 0.05 or *p* < 0.01). Furthermore, after calculating the total activities of all the seven bands above, a similar tendency was shown as individual activities: a marked increase of activities in TAA group at all the time points, extremely high at 1 month and 2 months (Fig. 5B. *p* < 0.05 or *p* < 0.01). Additionally, such an increase, whether individual or total activities, was also observed in TAA-D group (Fig. 5B. *p* < 0.05). By the way, since the active fragment of MMP-9 was observed in only one group, we didn’t calculate and analyze it.

### The activity of TIMP-1 in TAA-treated rat liver tissues

**Figure 6:**
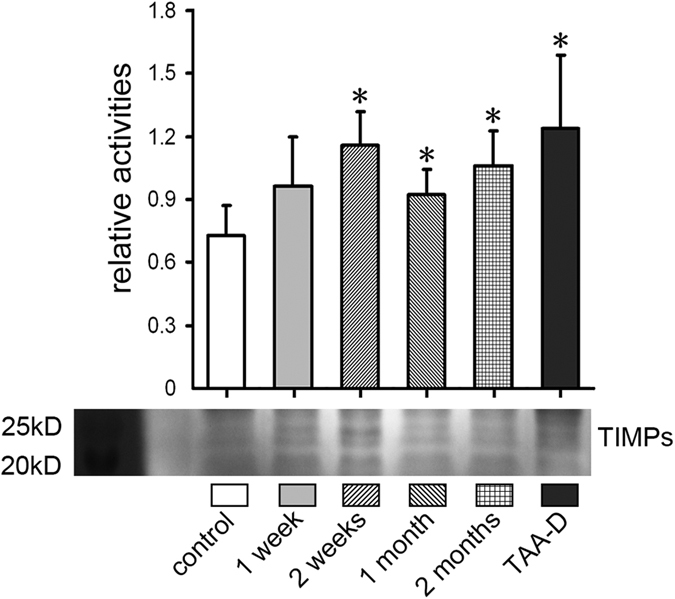
Activity of TIMPs in TAA-treated rat liver tissues. Activities of TIMPs by reverse gelatin zymography. n = 5; *p < 0.05 *vs.* control group.

To estimate the change of TIMP-1 during this experiment, the activity and level of TIMP-1 was analyzed by reverse gelatin zymography (Fig. 6), respectively. Compared with control group, the activity of TIMP-1 was increased in all time points but the increment was significant only at 2 weeks, 2 months and TAA-D (Fig. 6, *p* < 0.05).

### Up-expressed regulators of MMPs in TAA-treated rat liver tissues

**Figure 7:**
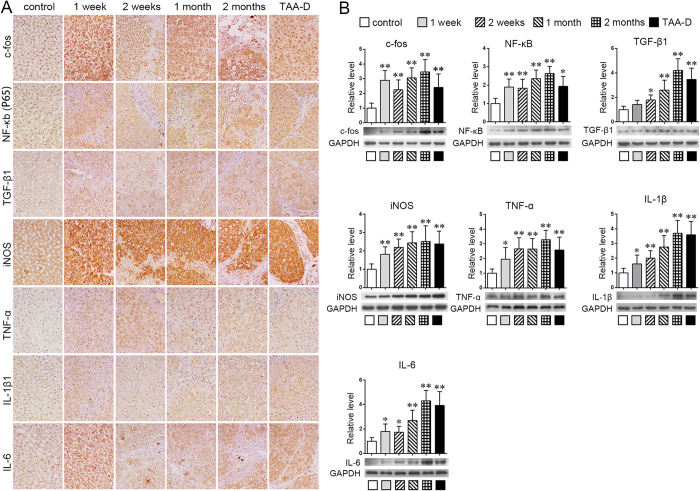
Over-expressed regulators of MMPs in TAA-treated rat liver tissues. **(A)** IHC results of iNOS, c-fos, NF-κb (P65), TGF-β1, TNF-α, IL-1β and IL-6 (×400 magnifications); **(B)** western blot results of iNOS, c-fos, NF-κb (P65), TGF-β1, TNF-α, IL-1β and IL-6. n = 5; *p < 0.05, **p < 0.01 *vs.* control group.

To determine the possible mechanism of over-actived MMP-2 and MMP-9, the expressions of some representative regulators of MMPs, including iNOS, c-fos, NF-κB (P65), TGF-β1, tumor necrosis factor α (TNF-α), interleukin 1β (IL-1β), and IL-6 were detected by IHC and western blot in TAA-treated rats (Fig. 7). As IHC results showed (Fig. 7A), it seemed the positive staining of nearly all of these factors were increased progressively from 1 week to 2 months, and stayed at high levels even in TAA-D group. Noticeably, iNOS, c-fos and IL-6 were increased acutely at the first time point, that is 1 week after TAA treatment. Additionally, western blot showed similar tendency (Fig. 7B, TAA group *vs.* control group, *p* < 0.05 or *p* < 0.01).

## Discussion

As early as 20 years ago, scientists noticed that the breakdown of ECM might play an important role during the progression of hepatic fibrosis^20^. After that, many reports exhibited the degradation of basement membrane constituents in hepatic fibrosis^21^,^22^. However, no systemic description was reported on the change of reticular fibers of ECM during hepatic fibrosis. While we went through the previous reports, we noticed that, as early as 1986, in a report on ethanol-induced liver fibrosis in rats by Tsukamoto, H. *et al*.^23^, an image delineated the degradation of reticular fibers in hepatic sinusoid, but such phenomenon was not discussed extensively in that report. In our research, after TAA treatment for 1 week (long before typical hepatic fibrosis), the degradation of reticular fibers in hepatic sinusoid of rats already occurred (Figs 1 and 3). Moreover, such degradation worsened as typical fibrosis developed. The decrease of reticular fibers caused by the degradation remained until one month after discontinuation of TAA administration (Figs 1 and 3). The similar pattern was also observed in BDL treated rats (Fig. 2). These results indicated that the breakdown of reticular fibers occurs at the early stage of the hepatic fibrosis, and furthermore, such degradation exists throughout the whole progression of hepatic fibrosis. Meanwhile, the similar pattern of decreased reticular fibers was shown in both TAA and BDL treatment, which proved that degradation of reticular fibers is a common event in both toxic and non-toxic induced hepatic fibrosis.

In addition, we found that the global area of reticular fibers rapidly enlarged after a period of time after the treatments were given (in both TAA and BDL). We hypothesized that the expansion was because of the increased reticular fibers inside of interlobular connective tissue, which is consistent with the classic pathological theory^24^.

MMPs are a group of ECM hydrolases, which are the essential part of regulating the synthesis and breakdown of liver ECM^25^. Tracing back to 1991, Herbst *et al*.^26^. reported the up-regulated mRNAs of MMPs in the early stage of toxic hepatic injury. In the year of 2000, Knittel *et al*.^27^ detected the up-regulated MMPs in early stage of hepatic injury. After these two reports, more and more investigations exhibited the increase of MMPs, which is an early event of hepatic injury, especially taking into account that a marked increase of MMPs could be found within a few hours after an insult^7^,^28^,^29^,^30^. These researches indicate that the damage of ECM might begin at the very early stage^31^. In our study, MMP-2 and MMP-9 were chosen to be detected, for they were well researched and are important components of MMPs family. We found that the expressions of MMP-2 and MMP-9 were at very low levels in normal liver, but were of high expressions in hepatocytes and cholangiocytes after rats were treated with TAA at all experimental time points (Fig. 4A). Moreover, by performing gelatin zymography, it was revealed that the activities of MMP complex, MMP-9, int-MMP-2, and active MMP-2 all significantly increased after TAA treatment (Fig. 5). Besides, such increase continued for 2 months during when the treatment was being given, and the increased activities remained at an abnormally high level even after discontinuation of TAA for a month (Fig. 5). Since we also observed that expressions of MMPs increased inside hepatocytes (Fig. 4A), it is reasonable to speculate that the reticular fibers along the hepatic cords might be collapsed firstly due to the high activities of MMPs.

The complicated regulation of MMPs occurs via transcriptional, post-transcriptional and post-translational mechanisms^16^,^17^,^32^,^33^.Key regulators of MMPs such as reactive oxygen species (ROS), NO, AP-1, NF-κB, TGF-β1 and inflammatory cytokines were highly increased in chronic injured and fibrotic liver according to previous reports^34^,^35^,^36^,^37^,^38^. In this research, several representative factors were analyzed to explain the up-regulation of MMPs (Fig. 7). Expressions of most MMPs are controlled at the level of transcription^16^,^33^. Transactivators such as AP-1 and NF-κB could interact with MMPs’ promoters and enhance their transcription^33^. TGF-β1 can either increase or inhibit MMP levels, depending on cell type^16^ and category of MMPs. For instance, MMP-9 contains TGF-β inhibitory elements, resulting in suppressed MMP expression^17^. Meanwhile, TGF-β1 also directly activates other transcription factors such as AP-1 and NF-κB, which implicate in the regulation of MMPs expression and enhance MMP promoters transactivity^33^,^39^, leading to increment of MMPs. In this research, c-fos (a member of AP-1), P65 (a member of NF-κB), and TGF-β1 were increased markedly during the initiation of hepatic fibrosis (Fig. 7), which might enhance the expression of MMP-2 and MMP-9. On the other hand, expression of MMPs was also controlled at the level of post-transcriptional modulation. For example, TGF-β increases MMP-2 and -9 levels, mainly by extending the half-life of MMP mRNAs^33^, while NO could increase the decay rate of the MMP-9 mRNA^40^ and active pro-MMP-2 and pro-MMP-9^16^,^41^. In this research, iNOS, an inducible isoform of nitric oxide synthases (NOSs), functioning to catalyze the production of nitric oxide, increased strongly in rat liver throughout TAA-treated process, which might play a role in the activation of MMP-2 and MMP-9. In addition, inflammatory cytokines, such as INF-α, IL-1β, and IL-6 all could up-regulate the expression and activation of MMPs^42^,^43^,^44^, which might also lead to the over-expression and activation of MMP-2 and MMP-9 according to our results (Fig. 7) and previous reports^37^.

On the other hand, it is also widely recognized that TIMPs, especially TIMP-1, play an important role in the progression of hepatic fibrosis^45^. TIMP-1 can reversibly bind to pro-MMP-9 and MMP-2 to down-regulate their activities^46^. Some researchers reported that TIMP-1 was markedly up-regulated in rat and human hepatic fibrosis^47^,^48^. It is also known that the increased TIMP-1 can inhibit the MMPs to slow down the degradation of ECM. In our experiment, the TIMP-1 was up-regulated after TAA treatment, but the extent of its up-regulation was lower than that of MMP-2 and MMP-9 (Fig. 6). As a result, the increase of TIMP-1 was relatively deficient and thus, could not protect the ECM from the degradation caused by overexpression of MMPs.

In human hepatic fibrosis, many previous studies reported the increase of MMP-2, MMP-9 and TIMP-1^49^,^50^. In our research, IHC was carried out on human cirrhotic liver tissue mircoarrays, and we found the expressions were also significantly high (Fig. 3B). And the positive staining was also widely observed inside of hepatocytes and cholangiocytes, very similar to the results in our animal experiment. Thus, we deduce that human hepatic cirrhosis might experience a similar pattern as rat hepatic fibrosis: reticular network along hepatic cords would be damaged and collapse during when hepatic cirrhosis develops.

In addition, our previous research, hepatocyte epithelial–mesenchymal transition (EMT) of TAA induced rat hepatic fibrosis, presented the decreased epithelial biomarkers and increased mesenchymal biomarkers of hepatocytes, which demonstrated that the hepatocytes undergo EMT during the late stage of hepatic fibrosis^19^. And it is known to us that the reticular network supplies the attachments to hepatocytes. Combining these knowledge and considering the results in the present research, the collapse of reticular network should be ahead of hepatocyte EMT, and in turn, we propose that the reticular fiber degradation might facilitate the hepatocyte EMT.

## Conclusions

The collapse of reticular fibers along hepatic cords occurs during the initiation and/or progression of hepatic fibrosis in both TAA-treated and BDL rats. Further more, by the stimulation of various highly expressed MMPs regulators, the up-regulation of MMP-2 and MMP-9 was likely responsible for this consequence. Such phenomenon could be considered as a marked pathological feature of the hepatic fibrosis progression, and also might be a new therapy approach to target hepatic fibrosis.

## Materials and Methods

The animal experiment was approved and conducted according to the regulations set by the Animal Use and Care Committee of Sichuan University. And all experiments were carried out in accordance with the manufacturer’s instructions.

### Animal experiment

Male Sprague Dawley (SD) rats were obtained from West China Medical Experimental Animal Center, Sichuan University. All the animals used in this study were kept under a 12 h light-dark cycles at a constant temperature and humidity with free access to chow and water. The animal procedure was approved by the Animal Use and Care Committee of Sichuan University and was conducted according to the regulations.

To induce hepatic fibrosis, thioacetamide (TAA) treatment and bile duct ligation (BDL) were carried out, respectively.

TAA treatment: TAA was injected peritoneally (TAA, Sigma Chemical Co., St. Louis, MO, USA; 250 mg/kg every 3 days). 55 male SD rats, weighing 200 ± 20 g, were randomized into control group (i.p. of physiological saline, 1 ml per rat/3 days), TAA group and TAA-discontinuance (TAA-D) group (TAA for 2 months, then discontinue TAA for 1 month). Time points to obtain specimens: one week, 2 weeks, 1 month and 2 months (specimens of TAA-D group were obtained for only one time point, i.e. 1 month after discontinuation of TAA treatment).

BDL: BDL surgery was performed following the steps of reference^51^. 36 male SD rats were divided into 6 groups according to the time points. Sham surgery was also performed to the control group. Time points: one day post-operation (dpo), 3 dpo, 7 dpo, 14 dpo, 21 dpo and 28 dpo.

Specimen obtainment: portions of liver tissues were fixed in 4% neutral buffered paraformaldehyde for histopathologic and immunohistochemical examinations, or immediately frozen in liquid nitrogen and stored at −80 °C for protein analysis.

### Histological stainings

Liver tissues were fixed in 4% neutral buffered paraformaldehyde, embedded in paraffin and sectioned (3 μm of thickness). Haematoxylin and eosin (HE) staining and Sirius red staining (0.1% Sirius red in saturated picric acid) were performed separately to evaluate the fibrotic level of liver tissues. Gomori’s reticular stain was preformed to identify reticular fibers. Five images per section (at ×100 and ×400 magnifications) from each rat were collected randomly, and Image-Pro Plus 6.0 software was used to evaluate the area of reticular fibers after the unspecific staining was eliminated.

### Immunohistochemistry (IHC) assessment

The paraffin sections of rat livers and human cirrhotic liver tissue mircoarrays (HLivH150CS03 and HLivH090PG01, Outdo Biotech Co.,Ltd. Shanghai, China) were deparaffinised and heated to 92–95 °C in 0.01 M/pH6.0 citrate buffer for 15 min followed by incubation with 3% H_2_O_2_ at 37 °C for 10 min. After blocking at room temperature for 20 min, the sections were incubated with primary antibodies (Supplementary Table 1) overnight at 4 °C followed by incubation with horseradish-peroxidase (HRP)-conjugated secondary antibody kits (SP-9001, SP-9002, SP-9003, ZSGB-BIO, Peking, China) at 37 °C for 30 min. Finally, the signals were detected using the Diaminobenzidine Substrate Kit (ZLI-9031, ZSGB-BIO), and positive staining was indicated by brown staining in the cytoplasm.

### Assessment of MMP-2, MMP-9 and TIMPs by gelatin zymography or reverse gelatin zymography

The gelatinolytic activities of intrahepatic MMP-2 and MMP-9 were investigated using zymography as described previously^20^. This assay can detect the active, prosome, active fragment and complex forms of gelatinases based on their different molecular weight. Frozen liver tissue was homogenized and whole proteins were extracted by ice-cold RIPA buffer (Beyotime, Shanghai, China). Equal amounts of proteins (20 μg) from each sample mixing with 2× SDS sample buffer (containing no reducing agent) were subjected to SDS–PAGE in an 8% SDS–PAGE gels containing 0.6 mg/ml gelatin (Sigma). After electrophoresis, the gels were removed and washed in a 2.5% Triton X-100 for 60 min (4 times × 15 min each) at room temperature to remove SDS from gels. Gels were incubated at 37 °C in incubation buffer containing 50 mM Tris–HCl, 5 mM CaCl_2_, 1 μM ZnCl_2_ and 0.02% Brij-35 at pH7.5 for about 48 h. The gels were stained with Coomassie Brilliant Blue R250 (0.25%) in 30% methanol and 10% acetic acid for about 4 hours, and then destained in 30% methanol and 10% acetic acid at room temperature over night to clearly visualize the digested bands. Proteolytic activities of MMP-2 and MMP-9 were visualized as clear bands against the blue background of stained gelatin. Proteolytic activities were normalized to total proteins in SDS-PAGE gels stained by Coomassie Brilliant Blue R250 (Supplementary Figure S5).

The anti-gelatinolytic activities of intrahepatic TIMPs (TIMP-1 and TIMP-3) were investigated using reverse gelatin zymography as described previously^52^. Main protocols were as same as gelatin zymography, 12% SDS–PAGE gels containing 0.1 μg/ml recombinant human Actived MMP-2 protein (ab174022, Abcam, PLC. Shanghai, China) and 0.6 mg/ml gelatin were applied. Anti-proteolytic activities of TIMPs were visualized as dark blue bands against the clear background of gel.

### Western blot analysis

Frozen liver tissue was homogenized and whole proteins were extracted by ice-cold RIPA buffer (Beyotime, Shanghai, China). Equal amounts of proteins (40 μg) from each sample were resolved by 10% or 12% SDS-PAGE, transferred to PVDF membrane (Millipore, Billerica, MA, USA) and blocked with 5% non-fat powdered milk in TBST (20 mM Tris–HCl pH 7.5, 150 mM NaCl and 0.1% Tween-20). Immunoreactive proteins, incubated with appropriate primary (Supplementary Table 1) and secondary antibodies, were visualized using ECL detection kit (Beyotime). Protein expression was normalized to GAPDH.

### Statistical analysis

All data were expressed as mean ± SD and analyzed by SPSS 21.0 software (SPSS, Chicago, IL, USA). One-way ANOVA and Post Hoc test were applied. The degrees of hepatic fibrosis were assessed by Ishak’s scoring system^53^. The presence of a statistically significant difference was denoted by *p* < 0.05.

## Additional Information

**How to cite this article**: Wen, S.-L. *et al*. Collapsed Reticular Network and its Possible Mechanism during the Initiation and/or Progression of Hepatic Fibrosis. *Sci. Rep.* **6**, 35426; doi: 10.1038/srep35426 (2016).

## Supplementary Material

Supplementary Information
