# Supplementary material for: Collapsed Reticular Network and its Possible Mechanism during the Initiation and/or Progression of Hepatic Fibrosis
Source: Sci Rep. 2016 Oct 14;6:35426. doi: 10.1038/srep35426 (PMC5064391; doi:10.1038/srep35426)
Supplement: Supplementary Information [file srep35426-s1.doc]

**Collapsed Reticular Network and its Possible Mechanism during the Initiation and/or Progression of Hepatic Fibrosis**

**Shi-Lei Wen^1#^, Shi Feng^1#^, Shi-Hang Tang^2^, Jin-Hang Gao^2,3^, Lin-hao Zhang^2,5^, Huan Tong^2^, Zhao-Ping Yan^2^, Ding-Zhi Fang^4*^**

*^1^Department of Human Anatomy, West China School of Preclinical and Forensic Medicine, Sichuan University, Chengdu 610041, Sichuan, PR China*

*^2^Department of Gastroenterology, West China Hospital, Sichuan University, Chengdu 610041, Sichuan, PR China*

*^3^Department of Peptides Related to Human Diseases, State Key Laboratory of Biotherapy, Sichuan University, Chengdu 610041, Sichuan, PR China*

*^4^ Department of Biochemistry and Molecular Biology, West China School of Preclinical and Forensic Medicine, Sichuan University, Chengdu 610041, Sichuan, PR China*

*^5^ West China School of Medicine, Sichuan University, Chengdu 610041, Sichuan, PR China*

^^ Shi-Lei Wen and Shi Feng contributed equally to this study.

**Correspondence*:

Prof. Ding-Zhi Fang,

E-mail: dzfang@scu.edu.cn;


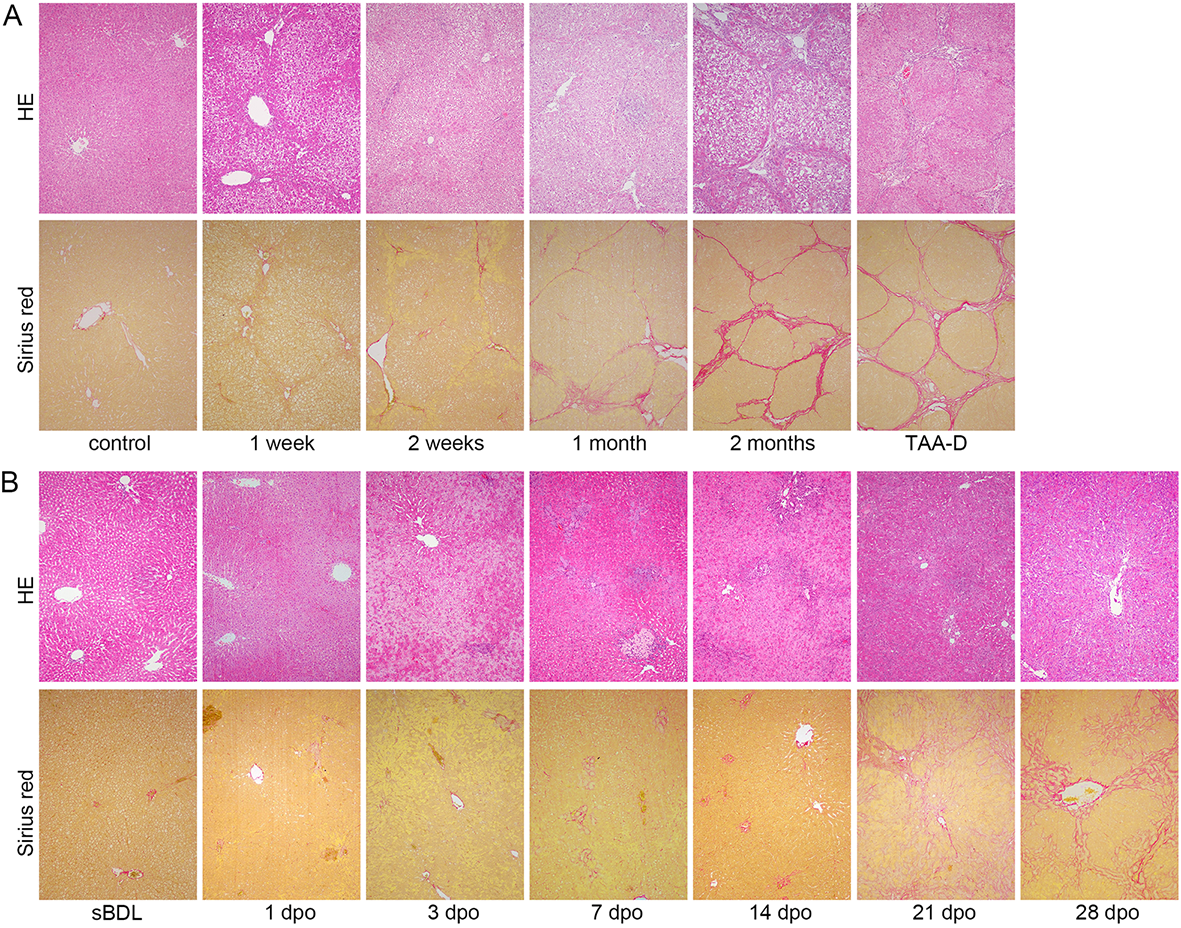


**Supplementary figure S1 HE and Sirius red stainings of rat liver tissues.** (**A**) HE and Sirius red stainings of TAA treated rat liver tissues; (**B**) HE and Sirius red stainings of BDL rat liver tissues. ×100.

**Supplementary Table 1**

**The antibody list for immunohistochemistry and western blot**

| **Antibodies** | **Dilutions**  **IHC/WB** | **Source** | **Catalog ＆ Company** |
| --- | --- | --- | --- |
| MMP-2 | 1:200/1:500 | Rabbit | 10373-2-AP, Proteintech Group, INC. Wuhan, China |
| MMP-9 | 1:250/1:500 | Rabbit | 10375-2-AP, Proteintech Group, INC. |
| TIMP-1 | 1:250/1:500 | Rabbit | 10753-1-AP, Proteintech Group, INC. |
| TGF-β1 | 1:200/1:500 | Rabbit | 18978-1-AP, Proteintech Group, INC. |
| p-Smad2/3(Ser 423/425) | 1:400/1:1000 | Goat | sc-11769-R, Santa Cruz Biotechnology |
| Collagen III | 1:1600/NA | Mouse | 22734-1-AP, Proteintech Group, INC. |
| IL-6 | 1:200/1:400 | Rabbit | 21865-1-AP, Proteintech Group, INC. |
| IL-1B | 1:200/1:400 | Mouse | 16806-1-AP, Proteintech Group, INC. |
| NF-kb(P65) | 1:200/1:500 | Mouse | Santa Cruz Biotechnology |
| iNOS | 1:200/1:500 | Rabbit | 18985-1-AP, Proteintech Group, INC. |
| TNF-α | 1:200/1:500 | Goat | 60291-1-Ig, Proteintech Group, INC. |
| c-fos | 1:200/1:500 | Goat | sc-52, Santa Cruz Biotechnology |
| GAPDH | 1:25000 | Mouse | 60004-1-Ig, Proteintech Group, INC. |

Abbreviations: IHC: immunohistochemistry; IL: interleukin; iNOS: inducible nitric oxide synthase; HRP: horseradish-peroxidase; MMP: matrix metalloproteinase; TNF-α, tumor necrosis factor-α; TGF-β1: transforming growth factor-β1; TIMP: tissue inhibitor of metalloproteinase.

**
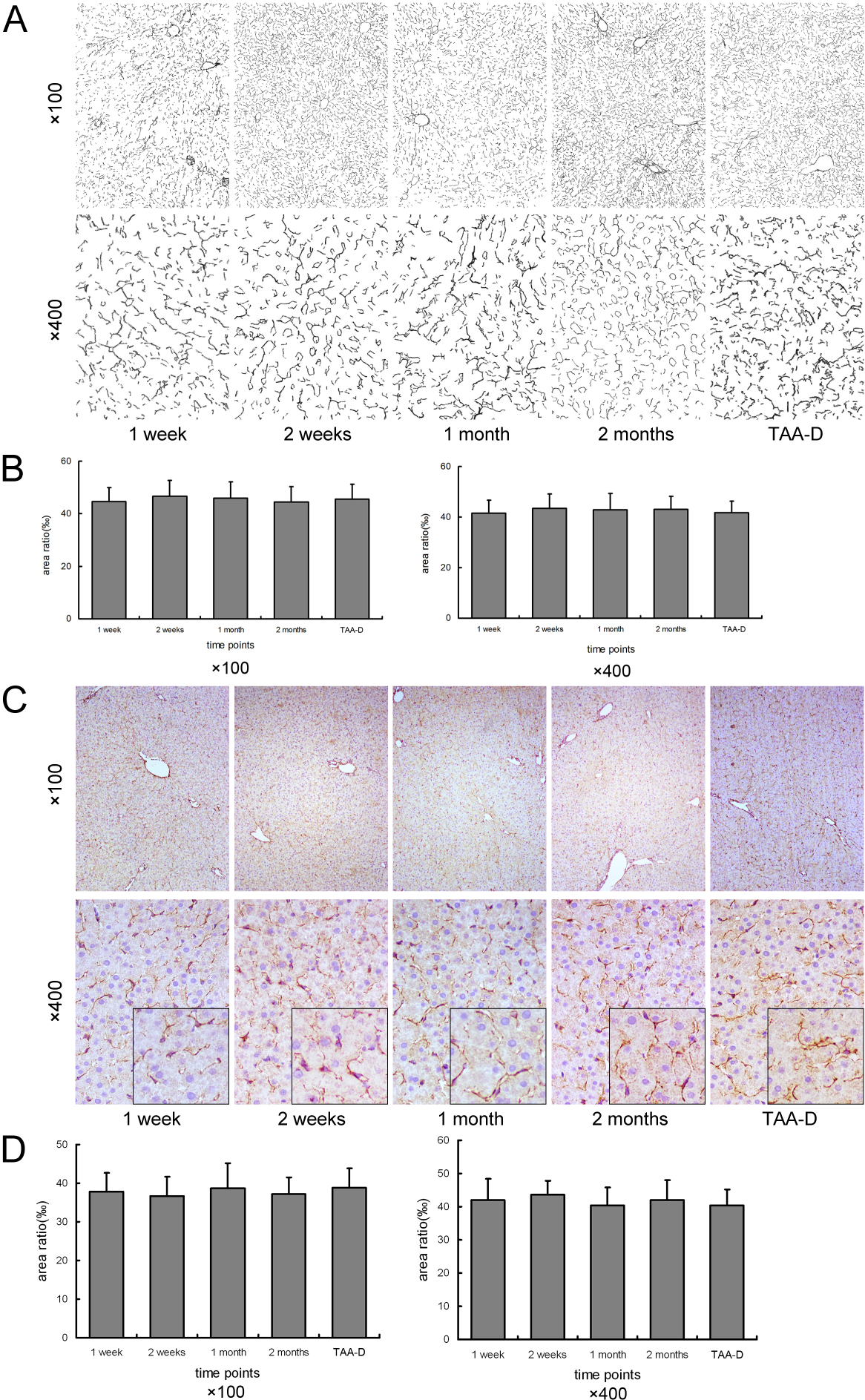
**

**Supplementary figure S2** **Reticular fiber stain and IHC of COL3A1 in control rat liver tissues time-matched for each TAA-treated rat.** (**A**) Reticular fiber stain result (The corresponding background was erased to show reticular fibers only); (**B**) Analyze results of reticular fiber area; (**C**) IHC result of COL3A1. insets×1000; (**D**) Analyze results of COL3A1 positive area. n=5; No significant difference showed up between each time points.


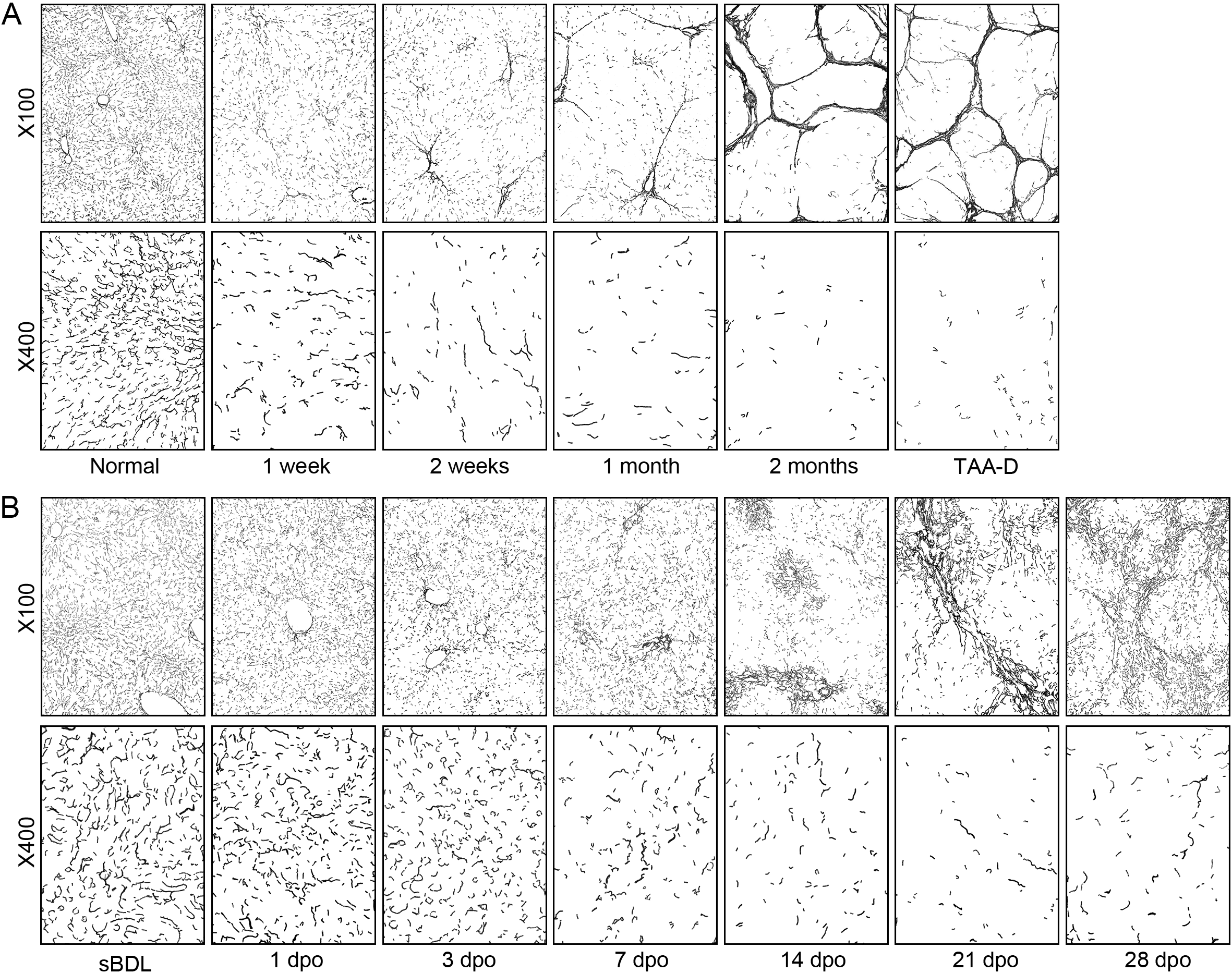


**Supplementary figure S3. Reticular fiber stain of TAA-treated (A) and BDL (B) rat liver tissues.** Gomori’s reticular fiber stain (The corresponding background was erased, showing reticular fibers only) ×100 and ×400.


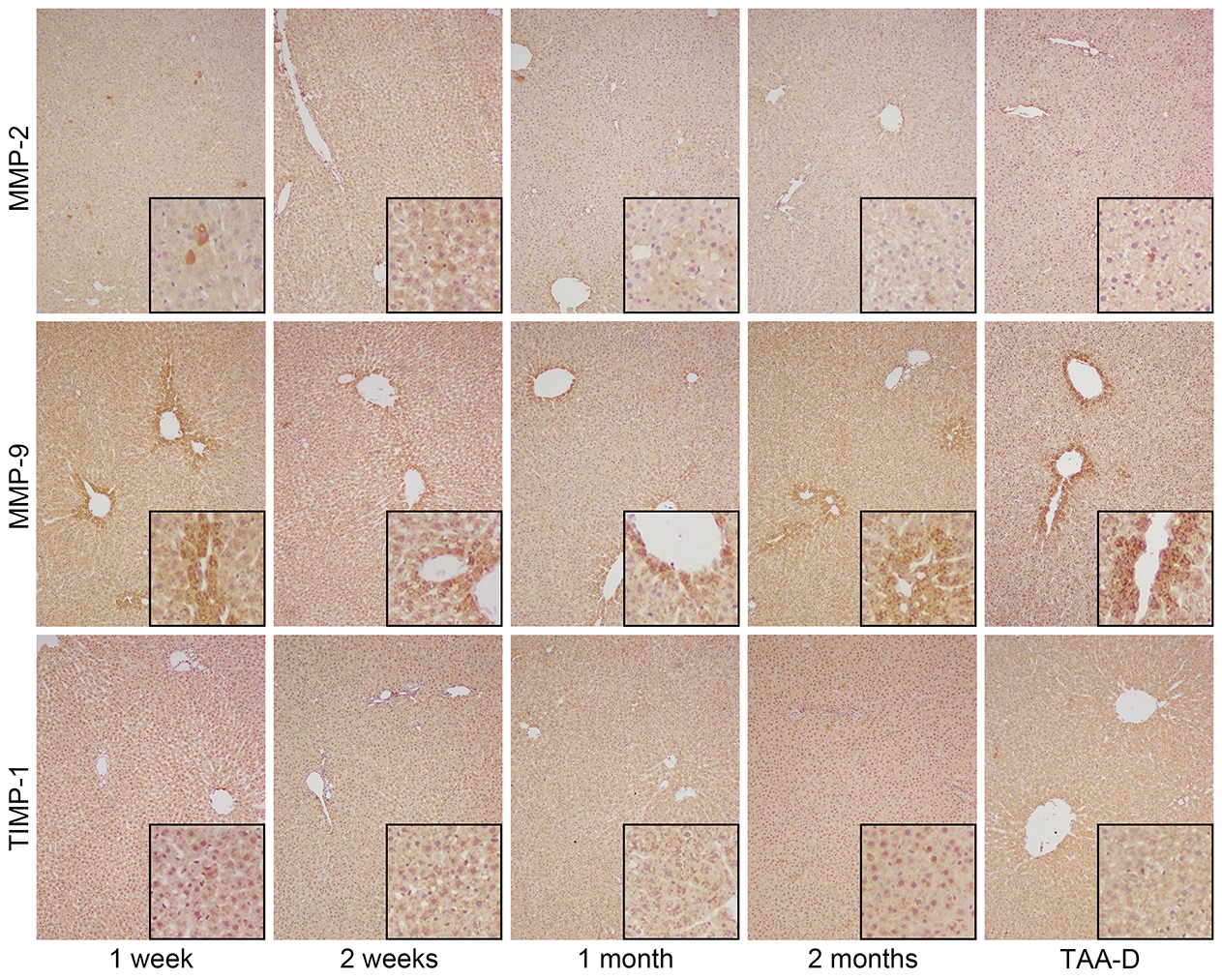


**Supplementary figure S4 IHC of MMP-2, MMP-9 and TIMP-1 in control rat liver tissues time-matched for each TAA-treated rat.** ×100.


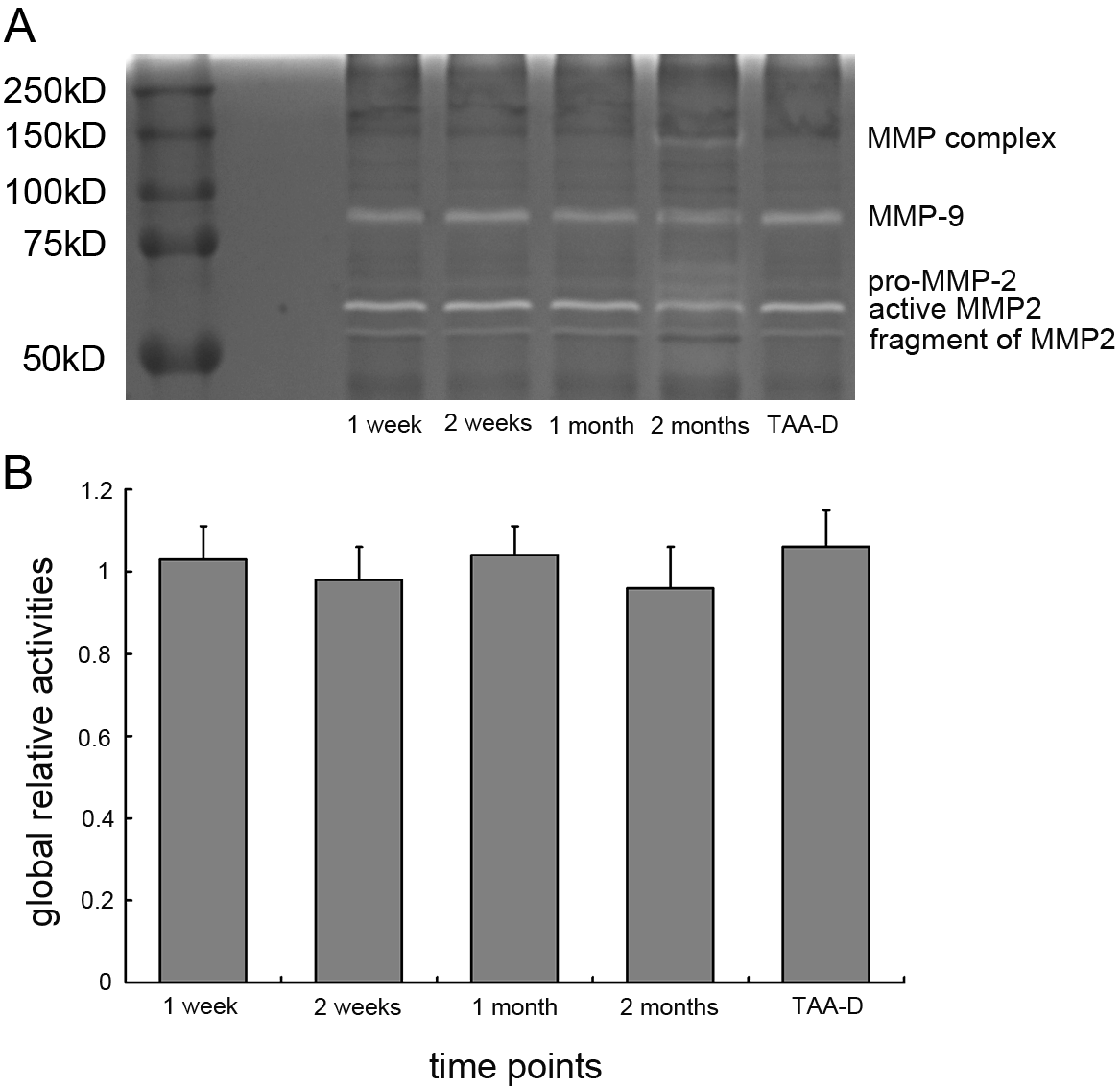


**Supplementary figure S5 Activities of MMP2 and MMP-9 in control rat liver tissues time-matched for each TAA-treated rat.** No significant difference showed up between each time points. (**A**) Activities of MMP-2 and MMP-9 by gelatin zymography; (**B**) Relative activities of total MMPs. n=5; No significant difference showed up between each time points.


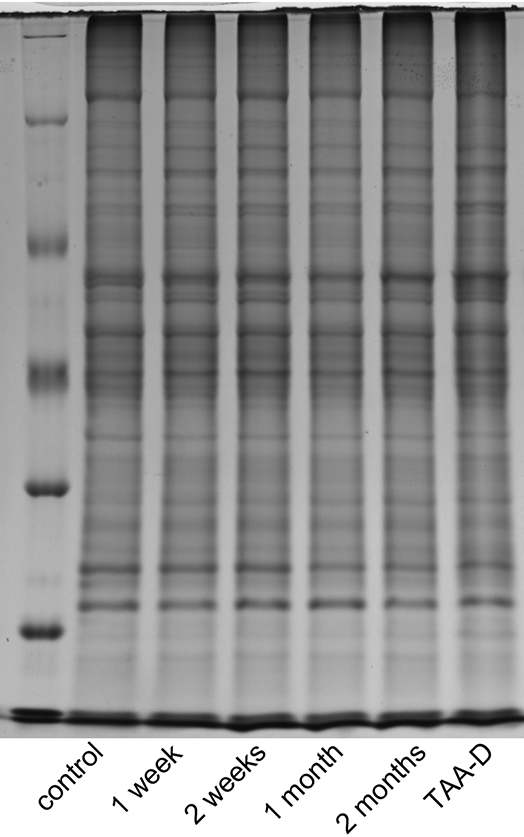


**Supplementary figure S6 Total proteins in SDS-PAGE gels stained by Coomassie Brilliant Blue R250.**
